# *Education:* An overview from the author of ‘Cefiderocol as rescue therapy for *Acinetobacter baumannii* and other carbapenem-resistant Gram-negative infections in intensive care unit patients’

**DOI:** 10.1093/jacamr/dlab056

**Published:** 2021-06-15

**Authors:** Marco Falcone

**Affiliations:** Infectious Diseases Unit, Department of Clinical and Experimental Medicine, University of Pisa, Pisa, Italy

## Abstract

This video is a presentation from Professor Marco Falcone from the University Hospital Pisa, Italy, originally presented at the BSAC conference ‘Into clinical practice: Meeting the challenges of Gram-negative infection management’ on 14 October 2020. Professor Falcone highlights the cefiderocol compassionate use case series that has been published in *Clinical Infectious Diseases* (Falcone M, Tiseo G, Nicastro M *et al.* Cefiderocol as rescue therapy for *Acinetobacter baumannii* and other carbapenem-resistant Gram-negative infections in intensive care unit patients. *Clin Infect Dis* 2020; doi: 10.1093/cid/ciaa1410). **The prescribing information for cefiderocol is available at: https://shionogi-eu-content.com/gb/fetcroja/pi.**

## Transparency declarations

This article is part of a promotional Supplement developed and sponsored by Shionogi B.V. The accompanying video formed part of an educational webinar for which the author received a speaking honorarium. The material underwent peer review by the Supplement Editors. Editorial assistance to Shionogi Europe was provided by Page Medical.

## Supplementary data

The video transcript is available as Supplementary data at *JAC-AMR* Online.

## Supplementary Material

dlab056_Supplementary_Data

