# Supplementary material for: Education: An overview from the author of ‘Cefiderocol as rescue therapy for Acinetobacter baumannii and other carbapenem-resistant Gram-negative infections in intensive care unit patients’
Source: JAC Antimicrob Resist. 2021 Jun 15;3(Suppl 1):i25. doi: 10.1093/jacamr/dlab056 (PMC8251248; doi:10.1093/jacamr/dlab056)
Supplement: dlab056_Supplementary_Data [file dlab056_Supplementary_Data.docx]

**Supplementary data**

**Video transcript**

I am Marco Falcone from the Division of Infectious Diseases, and I serve as an Associate Professor in Infectious Disease and an Infectious Disease Consultant at the University Hospital of Pisa in Italy. The goal of my presentation is to share our preliminary experience with the use of cefiderocol for the treatment of patients with severe nosocomial infection caused by carbapenem-resistant Gram-negative bacilli.

This first slide summarizes the epidemiological situation in my hospital, and it is really important to know the epidemiology of a carbapenem-resistant Gram-negative bacilli in order to analyse the resistance profile and to use the better therapeutic approach. As you see, we have a variable situation. Regarding Enterobacterales, for example, we have not one predominant mechanism of resistance. KPC is in blue, you see rectal colonized patients with rectal colonization, and in the yellow, patients who develop a bloodstream infection. We have a high prevalence of metallo-β-lactamases, NDM or VIM, and we also have a significant number of colonization and infection by *Acinetobacter baumannii*. This slide is not comprehensive—some species like *Pseudomonas* are not included—but is just an example to give you a picture of our epidemiological situation. This is characterized by multiple mechanisms of resistance in different species.

Here, I present our experience with the use of cefiderocol as rescue therapy for infection caused by *Acinetobacter baumannii* and other carbapenem-resistant Gram-negative infections. The paper was recently published in *Clinical Infectious Diseases*^1^ and we used this drug from the 1st of April to May 2020 in the context of compassionate use, with each prescription approved by our local ethics committee. Patients were selected if they had an infection by a CRE or non-fermenting Gram-negative bacilli resistant to carbapenems, were susceptible to cefiderocol and experienced clinical failure or serious adverse event from the previous antibiotic regimens. So, this therapy was not a first-line therapy but a second-line therapy in patients with no good clinical response to standard therapy. Cefiderocol was administered as a standard dose of 2 g every 8 hours in the 3 hour IV infusion, but we used a dosage of 2 g every 6 hours if the glomerular filtration rate was more than 120 at the DC recommended also in the CA RCP of the drug. To evaluate the impact of our therapy, we used clinical success as an endpoint; it was a composite endpoint, including the survival, resolution of signs and symptoms of infection and absence of recurrent microbiological failure. Microbiological failure was defined as the isolation of the same bacteria species of that first infection following more than 7 days from the discontinuation of cefiderocol.

Here is a description of our patient population. The first message is that our patients were patients with very severe underlying conditions. Half of the patients had COVID-19, where the patients were mechanically ventilated for SARS-CoV-2 pneumonia. And in the remaining half, most of them were burn patients—patients with extensive burn lesions of the body surface area. One patient was a post-surgical patient, who received a surgical intervention for colonic perforation. The age of the patients was variable, from 33 years old to two patients who were very old at 82 years old. But as you see also the SOFA score and APACHE II score were very high at the time of infection. They were severely ill, critical patients with very important underlying conditions.

The bacteria causing infection were *Acinetobacter baumannii* in six cases of bloodstream infection, and the majority of these cases of bloodstream infection were in burn patients. You can also see the MIC of cefiderocol—there was full susceptibility in all cases. We also treated the four cases of ventilator-associated pneumonia which, as you know, is a very difficult infection to treat. And these cases of ventilator-associated pneumonia were caused by mixed infections; for example, NDM-producing *Klebsiella* plus *Stenotrophomonas*, NDM alone in one case, *Acinetobacter* plus NDM in another case, and *Acinetobacter* alone in one case.

With regards to the initial antibiotic regimen, it was, in the majority of cases, a colistin-based regimen. Colistin was variably associated with other drugs including tigecycline, fosfomycin, meropenem, ampicillin/sulbactam or other drugs. Despite treatment with the antibiotic therapy, in 70% of cases there was clinical failure or persistence of fever or positive culture. In 30%, we observed severe renal or hepatic toxicity.

The clinical outcomes of our patients are shown here. The majority of them, 9 out of 10, received monotherapy with cefiderocol and 2 were in continuous renal replacement therapy. The clinical success was 70% (7 out of 10), and the 30 day mortality after the infection was treated with cefiderocol was 10%. When you consider the severity of underlying conditions, the fact that patients were all in ICU and the severity of the *Acinetobacter baumannii* infection, we see this data as quite positive. The microbiological outcomes are very important since we observed a microbiological failure in two patients; we define microbiological failure as a relapse—that is a new culture by the same strain after the discontinuation of cefiderocol.

What I want to underline is that both patients were burn patients, had a prolonged ICU stay and were colonized on the skin by *Acinetobacter baumannii*. Burn patients are very particular kinds of patients and when the pathogen colonizes or infects the skin, it is very difficult to eradicate. You can treat the acute infection, but the patient still remains colonized. It’s also very common for the same strain to cause a relapsing infection. What we also have to take into consideration is that in one of the cases we isolated a strain with an increased MIC for cefiderocol—from 0.25 to 4. The strain was formerly resistant in the second episode and this should be investigated because it may be that the patients had a focus of infection which was not removable or have persistent chronic colonization by the same strain—these are conditions that promote and favour the selection of antibiotic-resistant strains. However, no breakthrough infection was observed during the cefiderocol treatment and the median time to microbiological failure was 21 days.

This slide shows the microbiological tests that we performed on 21 strains. I selected them mostly from patients in our hospital that were treated with cefiderocol, otherwise from those that we would try to treat with cefiderocol. The susceptibility overall was 91%, just one isolate of *Acinetobacter baumannii* and one isolate of NDM *Klebsiella* that were resistant to cefiderocol was observed. This is an expected result because for metallo-β-lactamases and Enterobacterales, our MICs are very close to the breakpoint and this may impact susceptibility to cefiderocol. To conclude, almost all strains were susceptible.

My last slide shows the additional unpublished cases that were treated with cefiderocol and that for who we have a 30 day follow-up after the start of therapy. There are other patients that were also in therapy, however for these we have no 30 day follow-up.

As previously mentioned, we treated six additional patients. All were ICU patients with severe conditions like the previous 10 that I discussed in the publication—burns, abdominal perforation, COVID-19 and cerebral haemorrhage. Infections were bloodstream infections, complicated intra-abdominal infection, bloodstream infection by *Acinetobacter baumannii* and ventilator-associated pneumonia of *Acinetobacter baumannii*.

Here are the data I summarized of 16 patients, of which I published 10 and left 6 unpublished. Our results showed that clinical success was up to about 70% and 30 day mortality was 12.5%. I remarked these results because in *Acinetobacter baumannii* bacteraemia infections in ICU patients of this type, the 30 day mortality is usually higher than what we observed here.

When considering the 25% of microbiological failure, I want to remark that microbiological failure was also observed in another burn patient. I think that burn patients are a particularly distinct patient population that are quite different to ICU patients in general and are probably more predisposed to remain colonized by some resistant microorganisms and are more predisposed to have a relapse.

These data are very useful for us because we had a good possibility to treat the patients where we have several difficulties to manage such as monitoring colistin for renal toxicity or combining with other drugs, for example tigecycline or other bacteriostatic drugs; if you are treating a severe infection, these difficulties cause a problem. We hope that future studies will confirm our results on cefiderocol, and that we will be able to use this new option to better treat our severely ill ICU patients.

References

**1**. Falcone M, Tiseo G, Nicastro M *et al.* Cefiderocol as rescue therapy for *Acinetobacter baumannii* and other carbapenem-resistant Gram-negative infections in ICU patients. *Clin Infect Dis* 2020; doi:10.1093/cid/ciaa1410.
